# Multisystem Inflammatory Syndrome of a Neonate From a COVID-19-Infected Mother: A Case Report

**DOI:** 10.7759/cureus.23046

**Published:** 2022-03-10

**Authors:** Abdul Malek, Mukesh Khadga, Md Nurnobi Zahid, Sanjida Mojib, Reena Debnath, Sheela Khan, Mainul Haque, Brian Godman, Salequl Islam

**Affiliations:** 1 Pediatrics, GreenLife Medical College Hospital, Dhaka, BGD; 2 Pediatrics, Newborn Intensive Care Unit (NICU), GreenLife Medical College Hospital, Dhaka, BGD; 3 Community Medicine, GreenLife Medical College, Dhaka, BGD; 4 Pharmacology and Therapeutics, National Defence University of Malaysia, Kuala Lumpur, MYS; 5 Centre of Medical and Bio allied Health Sciences Research, Ajman University, Ajman, ARE; 6 Pharmacoepidemiology, Strathclyde Institute of Pharmacy and Biomedical Sciences, Glasgow, GBR; 7 School of Pharmacy, Sefako Makgatho Health Sciences University, Pretoria, ZAF; 8 Microbiology, Jahangirnagar University, Dhaka, BGD

**Keywords:** multisystem inflammatory syndrome, sars-cov-2, rt-pcr, neonate, covid-19

## Abstract

In neonates, the prevalence of severe acute respiratory syndrome coronavirus 2 (SARS-CoV-2 - COVID-19) is lower. There is the potential for vertical transmission of SARS-CoV-2. To date, only a few reports suggest this possibility. Neonates usually have mild symptoms, but some develop multisystem involvement, which is a concern. COVID-19 infections have been reported both in pregnant women and their neonates. However, the evidence of vertical or horizontal transmission modes has not been fully established. We recorded a case study where a 33-year-old mother was tested positive for COVID-19 infection by RT-PCR during her 27^th^ week of gestation and needed ventilator support for her respiratory distress at that time for 11 days. Subsequently, she gave birth to a female baby at the 35^th^ week via a lower uterine segment cesarean section. The neonate manifested a severe multisystem inflammatory syndrome associated with her possible COVID-19 infection. Sharing her uncommon clinical presentation, immunological syndrome, and disease outcome are noteworthy for similar unforeseen pediatric case management to help guide future investigations and care.

## Introduction

Severe acute respiratory syndrome coronavirus 2 (SARS-CoV-2 [also known as COVID-19]) infection during pregnancy may increase the risk of stillbirth, neonatal death, low birth weight, preterm birth, fetal distress, and perinatal asphyxia [1]. Out of COVID-19 confirmed cases, globally on June 14, 2021, 2.7% were less than 10 years of age [2]. Furthermore, the COVID-19 infection is not as common below one year of age; thereby, the projected incidence rate among this age group was 12%-18% [3]. The disease has a milder course in children than adults and may present with non-specific symptoms, especially in neonates that are not clearly explained [4-7]. However, multiple studies reported that the issue is possibly associated with the low availability of angiotensin-converting enzyme 2 (ACE2) receptors among children below one year of age [8-10]. ACE2 receptors are determined as the principal access point of COVID-19 in the human system [11-13]. Alongside this, the perinatal vertical transmission of SARS-CoV-2 appears rare [14-16]. The low risk for vertical transmission could potentially be explained by the low placental expression of canonical receptors, with negligible co-transcription of ACE2 and transmembrane protease serine 2 (TMPRSS2) in the placenta, which are necessary for the virus entry [17]. The SARS-CoV-2 virus is a rare cause of fetal inflammatory response syndrome (FIRS) [18] and can be associated with multisystem inflammatory syndrome in children (MIS-C) [19]. However, in Bangladesh, Kawasaki disease (KD)-like symptoms/hyperinflammatory syndromes are a real possibility among children admitted with COVID-19 across countries [20]. This is a concern, primarily as such children, including neonates, may be managed in pediatric intensive care units (ICUs), with mortality in such units higher in lower- and middle-income countries than higher-income countries [21,22].

## Case presentation

At the 27^th^ week of gestation, a 33-year-old female (gravida-3rd, para-0+2) with fever and cough for three days and also associated with mild respiratory difficulty was admitted to Green Life Hospital on October 7, 2020. She was tested positive for COVID-19 by real-time polymerase chain reaction (RT-PCR) of the nasopharyngeal and oropharyngeal swab. The chest radiograph showed patchy consolidation. Her respiratory distress increased on October 10, and she was moved to the ICU and kept on ventilator support for 11 days. Her respiratory distress subsided by 11 days, and she was subsequently transferred to the appropriate unit of the hospital as per patient need demand after 12 days of treatment at ICU. Later, she was discharged from the hospital with proper advice and medication needed to do at home [12]. The fetus in the utero was reasonably well throughout her hospital stays.

The pregnant woman had a history of overt diabetes mellitus (DM), hypothyroidism, and pregnancy-induced hypertension (PIH). She was prescribed short and long-acting insulin, levothyroxine, and anti-hypertensive drugs. At the 34^th ^week of gestation, she developed a sudden rise in blood pressure (BP) with increasing frequency of micturition. Later she was diagnosed with a urinary tract infection (UTI). Despite all possible treatments, the patient's BP was not maintained. A medical board decided to terminate the pregnancy through the lower uterine segment cesarean section (LUCS) at the 35^th^ week of gestation.

On December 7, 2020, at 4.30 pm, an emergency cesarean section was performed, and a female baby was delivered weighing 1,950 grams. The baby cried immediately after birth, and APGAR (Appearance, Pulse, Grimace, Activity, and Respiration) score was 8/10 in both the first and fifth minute, respectively, and required only routine resuscitation. Chlorhexidine (Hexicord) solution was applied over the umbilical stump, and I/M Vitamin K1 (phytonadione) was given. The baby was normothermic (temperature 36.5°C), euglycemic, reflexes and activities were good, heart rate was 140 beats/min, respiratory rate was 60 breaths/min. The baby was isolated from her mother immediately after birth without skin-to-skin contact. At postnatal age two hours, the baby developed respiratory distress in the form of tachypnea, chest indrawing, grunting with peripheral cyanosis. Consequently, the baby was moved to the neonatal ICU (NICU) with proper wrapping. The baby was subsequently managed by ensuring thermal care, was given nothing per oral (NPO), put on continuous positive airway pressure (CPAP, Down's score 4), and treated with injectable antibiotics [parenteral ampicillin (50 mg/kg per dose 12 hourly) and gentamicin (5 mg/kg per dose daily) after sending an infected sample for the specific laboratory analysis.

An investigation with a nasal and oropharyngeal swab for RT-PCR for COVID-19 was also undertaken with planning for echocardiography and electrocardiography (ECG) and a chest x-ray. The RT-PCR appeared negative. C-reactive protein (CRP) did not go up at the beginning, but a complete blood count (CBC) showed neutrophilia and lymphopenia along with 1% of eosinophils, which indicated the probable presence of viral infection. Other parameters in the CBC were within the normal range. The electrocardiography showed moderate persistent pulmonary hypertension in the neonate (PPHN) with the pulmonary artery systolic pressure (PASP) at 49 mm of Hg. The echocardiogram report further indicated moderate perimembranous ventricular septal defects (VSDs) with a small patent ductus arteriosus (PDA) and a small atrial septal defect (ASD); however, the chest x-ray was found within normal range. Empirically, Ceftazidime and Amikacin were started as treatment along with pulmonary vasodilator, Sildenafil, and I/V Frusemide on day 7.

Nasogastric (NG) tube feeding was started on day 2 of the postnatal period with I/V calcium supplements for hypocalcemia (6.2 mg/dL). Serum creatinine was high (1.3 mg/dL); consequently, the antibiotics' dose was adjusted according to the renal values. Phototherapy was started empirically as the neonate was found to be icteric. Blood culture revealed no microbial growth.

The CRP levels were checked on regular intervals, increased at three days of age, and remained high until 18 days. Procalcitonin was measured and found within the normal range. Her platelet count decreased to 85×10^9^/L. Total serum bilirubin was 18 mg/dL, with serum creatinine becoming normal (0.6 mg/dL) with normal electrolyte levels. Antibiotics were subsequently changed to injection meropenem and vancomycin from sepsis prediction. However, the second-cycle blood culture further revealed no bacterial growth. The second-round nasopharyngeal sample was examined for COVID-19 by RT-PCR, the result appeared negative again.

Hypocalcemia still persisted (6.2 mg/dL) until day 6 and D-dimer became elevated (2.4 µg/mL) with normal serum ferritin (137 ng/mL) level. The medical board thought of anticoagulant therapy or steroid therapy as an alternative. Neither of the two therapies was given as the platelet count went seriously low (below 20,000 to 45,000/mm^3^). The second electrography showed closed ducts with resolved PPHN with remaining perimembranous VSD and small ASD with mild valvular pulmonary artery stenosis. Thereupon, we stopped Sildenafil and I/V Frusemide on day 12 and started oral Frusemide.

The neonates' prothrombin time (PT) was increased (20.8 sec) with an international normalized ratio (INR) at 1.59, and the activated partial thromboplastin time (APTT) levels were as high as 128.2 sec. PT and APTT remained elevated for 18 days. Fresh frozen plasma (FFP) transfusion was given for coagulopathy. Serum glutamic pyruvic transaminase (SGPT) levels were normal. The baby remained normothermic and euglycemic throughout those events.

We gradually decreased the continuous positive airway pressure (CPAP) support and oxygen support of the neonate as her respiratory distress improved day by day. A breastfeeding trial along with dropper feeding was undertaken, and subsequently, kangaroo mother care (KMC) was initiated.

On postnatal age day 11, CRP was raised to 102.1 mg/L, and serum IL-6 was found to be high (13.48 pg/mL), suggestive of post-COVID-19 effects. Serum albumin was lower than the normal range (31 g/L). Intravenous immune globulin (IVIG, Pentaglobin, Biotest AG, Germany) was given for consecutive five days. As per the National Neonatal Septicemia Management Guideline, I/V Fluconazole was also started after day 14 of the hospital stay [23-26].

Serum Ca2+ level became normal, and I/V calcium gluconate was stopped with the continuation of breastfeeding. The patient's Hb level decreased to 9.8 g/dL, for which packed cell transfusion was given once. Other diagnostic parameters improved, including the platelet count raised to 300×10^9^/L, CRP came down to 24.2 mg/L, PT decreased to 16.1 sec.

Clinically, the baby became hemodynamically stable at day 20 and free from the external oxygen need. Thereby, the patient was shifted from NICU to the general baby cot with her mother for further observation. We discharged the baby with a weight of 2.1 kg on December 31, 2020 at her postnatal age of 24 days without any abnormal severe clinical symptoms.

The medical board advised retinopathy of prematurity (ROP) screening, developmental, hearing assessment, and also for regular follow-up for cardiac problems. We further recommended immunization according to the expanded program on immunization (EPI, a program of vaccination of Bangladesh government against 10 childhood diseases - diphtheria, pertussis [whooping cough], tetanus, tuberculosis, polio, measles, and polio, pneumococcal pneumonia, hepatitis B, Hemophilus influenzae, rubella) schedule and next follow up after two weeks from discharged date.

## Discussion

This case study reported clinical presentation and immune response of a multisystem inflammatory syndrome (MIS), also called pediatric inflammatory, multisystem syndrome (PIMS), of a neonate born from a COVID-19-infected mother. The report has described the treatment undertaken for this MIS/PIMS, its rationale, and progressive treatment outcomes. This case presented a premature birth and respiratory distress (tachypnoea, tachycardia, chest indrawing, grunting with peripheral cyanosis). No gastrointestinal symptoms were seen in our case. Neonates and children infected with COVID-19 may have variable presentations ranging from asymptomatic infection to severe respiratory distress [4]. The neonatal presentation can have a history and symptoms of prematurity, known contacts with COVID-19 patients, respiratory distress, poor feeding, lethargy, diarrhea, and vomiting [27].

COVID-19 presentations in pregnant women are different and complicated based on the various organ system affected [28]. This case study has described some distinct clinical manifestations during pregnancy and post-delivery. COVID-19 infection to neonates or infants is less frequent [14]. However, reported in multiple studies from all over the globe [15,29], which has attested the possible linkage to the clinical presentation of our case neonate. The neonate case has shown a mild course of the disease at the beginning and then progressed to the potentially life-threatening condition, known as MIS-C or PIMS-TS [16,30-32]. A lower severity of COVID-19 in neonates at the beginning of the infection can be explained by the lower expression of the ACE-2 receptors or their functionally immature structure in neonatal mucosa SARS-CoV-2 binds to start the disease [27,33]. Consequently, these neonates must be regularly monitored to ensure appropriate care. Oxidative stress (OE) was reported in the pathophysiology of MIS-C, similar to the KD presentation [34,35].

COVID-19 patients have average or decreased WBC count with reduced lymphocyte, and normal or increased CRP [36]. The rise of procalcitonin is usually expected in the majority of cases. Severe cases may have increased D-Dimer, PT, APTT, and IL-6 [37]. Chest x-ray findings are non-specific, including consolidation, ground-glass opacity, pneumothorax, or normal [38]. Our patient had an average total WBC count, but neutrophilia, lymphopenia, and eosinopenia were present. Procalcitonin and chest x-ray findings were normal in our case, but D-Dimer, PT, APTT, and IL-6 were increased. Our results align with some recent previous reports [39,40]. An electrography may show severe biventricular dysfunction with decreased left ventricular ejection fraction and global hypokinesia [41]. In our case, the echocardiography showed moderate PPHN (PASP 49 mm of Hg) with moderate perimembranous VSD and small PDA and small ASD, which were later corrected by medications.

There are published reports which show vertical transmission of COVID-19 infection [42,43]. Although our case was tested negative twice for COVID-19 disease through the RT-PCR test, the clinical and biochemical parameters strongly suggest COVID-19 infection. A Septic workup was ruled out in our case. The vertical transmission was confirmed through virological testing and the presence in the placenta [42]. The main treatment options are supportive and symptomatic treatment, oxygen therapy, and mechanical ventilation. In severe cases, corticosteroids are found to be beneficial [42]. We did not give steroids in our case because of thrombocytopenia. Our patient was managed in the line of neonatal sepsis and was discharged when the baby was stable as per discharge criteria. As a result, antibiotic treatment was initiated. We are aware of the overuse of antibiotics in children with COVID-19 across countries, including Bangladesh adding to antimicrobial resistance, with prudence advocated in Pediatric guidelines, including Bangladesh [20,44,45]. However, we felt this was appropriate in this situation.

## Conclusions

We describe an uncommon case of MIS-C-like illness in a neonate born from a COVID-19-positive mother. The clinical presentation of the neonate was similar to KD and COVID-19 symptoms. Standard treatment for children with MIS-C is IVIG, aspirin, and steroids; however, the simultaneous COVID-19 linkage complicated treatment choice, including steroids and antibiotics for possible neonatal sepsis. The treatment regimen and management strategy may help the medical community find beneficial treatments of neonatal COVID-19 infection and MIS-C as more neonates and children are admitted to hospitals worldwide with COVID-19.
